# *PIK3CA* mutations and their impact on survival outcomes of patients with endometrial cancer: A systematic review and meta-analysis

**DOI:** 10.1371/journal.pone.0283203

**Published:** 2023-03-21

**Authors:** Hanna K. Bredin, Camilla Krakstad, Erling A. Hoivik

**Affiliations:** 1 Department of Clinical Science, Centre for Cancer Biomarkers, University of Bergen, Bergen, Norway; 2 Department of Obstetrics and Gynecology, Haukeland University Hospital, Bergen, Norway; Teikyo University, School of Medicine, JAPAN

## Abstract

Several studies have highlighted the frequent alterations of the PI3K pathway in endometrial cancer leading to increased signaling activation with potential for targeted treatment. The objective of this meta-study was to evaluate how *PIK3CA* exon 9/20 mutations affect survival in endometrial cancer patients, based on available literature. Topic-based search strategies were applied to databases including CENTRAL, MEDLINE, Embase, Web of Science and COSMIC. All studies assessing the impact of mutations in exon 9 and exon 20 of *PIK3CA* on survival rates of endometrial cancer patients were selected for inclusion. Statistical meta-analysis was performed with the ‘*meta’* package in RStudio. Overall, 7 of 612 screened articles were included in the present study, comprising 1098 women with endometrial cancer. Meta-analysis revealed a tendency of impaired survival for patients with *PIK3CA* exon 9 and/or exon 20 mutations (RR 1.28; 95% CI 0.84, 1.94; p = 0.25). This tendency was consistent in subgroup analyses stratified by histologic type or -grade, with the most prominent effect in low-grade endometrial cancers (RR 2.04; 95% CI 0.90, 4.62; p = 0.09). In summary, these results suggest that *PIK3CA* mutations negatively influence survival outcomes of patients with endometrial cancer, including those with low-grade tumors.

## Introduction

Endometrial cancer (EC) is the most frequent pelvic malignancy among women in countries with a high human developmental index [1]. Obesity and high age are important risk factors for the disease, and with rising obesity rates and an aging world population, the number of patients with EC will increase by over 45% worldwide by 2040, along with the associated mortality [1].

ECs has traditionally been classified into type I or type II based on histological characteristics, grade and hormone receptor expression [2]. As recent molecular studies have further revealed disease heterogeneity, this dualistic classification of EC is now supplemented by four distinct molecular subgroups with disparate prognosis: POLE mutated (ultra-mutated), microsatellite instable (hypermutated), copy-number-low (microsatellite stable) and copy-number-high (serous-like) [3]. Accordingly, more accurate diagnosis, prognostic prediction and individualized treatment of EC is further augmented by genomic and molecular studies. Despite this, areas of uncertainty remain, including how mutations in *PIK3CA*, the second most altered gene in EC, affect patient survival.

*PIK3CA* encodes the p110α protein, the catalytic subunit of the phosphatidylinositol-3-kinase (PI3K) enzyme which is a key component in the PI3K signaling pathway. This pathway is often altered in solid cancers, most commonly in epithelial cancers, with the most frequent aberration rate in EC [4, 5]. The pathway is critical in the regulation of cell survival, proliferation and apoptosis, and dysregulation is associated with carcinogenesis [4]. This has made the PI3K pathway an attractive therapeutic target, and evaluation of dual PI3K/mTOR inhibitors, pan-PI3K inhibitors, and isoform-specific inhibitors in combination therapy have shown promising results in clinical trials, also in EC [6–12].

The reported overall *PIK3CA* mutation frequency in EC is ranging from 12–39% by Sanger sequencing [13–18] and 50% according to next generation sequencing (NGS) [19] with differences likely due to regions covered and sequencing methodologies applied. Mutations of *PIK3CA* cluster at hotspot sites, and in EC 80% of the mutations are located in the helical (amino acid residues p.E542, p.E545 and p.E546) and kinase domains (amino acid residue p.H1047), corresponding to exon 9 and exon 20, respectively [15]. Most *PIK3CA* mutations lead to a constitutive active PI3K pathway, but interestingly, the literature is inconsistent with regards to the impact of *PIK3CA* mutations on patient survival across many cancer types [20–24]. Also in EC, findings are contradictory, and have suggested that *PIK3CA* mutations are favorable [25], unfavorable [13, 18] or have neutral effect on patient survival [13, 26, 27]. Understanding the role of *PIK3CA* mutations in relation to clinical survival in endometrial cancer is essential for further development of selective p110a inhibitors, clinical trials and therapeutic approaches. We therefore aimed to investigate the association between *PIK3CA* exon 9/20 mutations with survival outcomes in patients with EC through a systematic review and meta-analysis.

## Materials and methods

The present meta-study was designed according to the Preferred Reporting Items for Systematic Reviews and Meta-Analyses (PRISMA) guidelines [28]. The study was based on review of data that have already been published, and therefore patient consent and institutional review board approval were not required. The study is registered with PROSPERO (International Prospective Register of Systematic reviews) ID CRD42021230618.

### Types of studies and patients

The eligibility criteria for the inclusion of studies were predetermined. All observational studies (prospective, retrospective and case series) that assessed the impact of mutations in exon 9 and/or exon 20 of the *PIK3CA* gene on survival rates (disease-specific survival [DSS] and/or overall survival [OS]) of EC patients were selected for inclusion. Studies were selected irrespective of the stage of disease and histological subtype.

### Information sources and search methods

**Fig 1:**
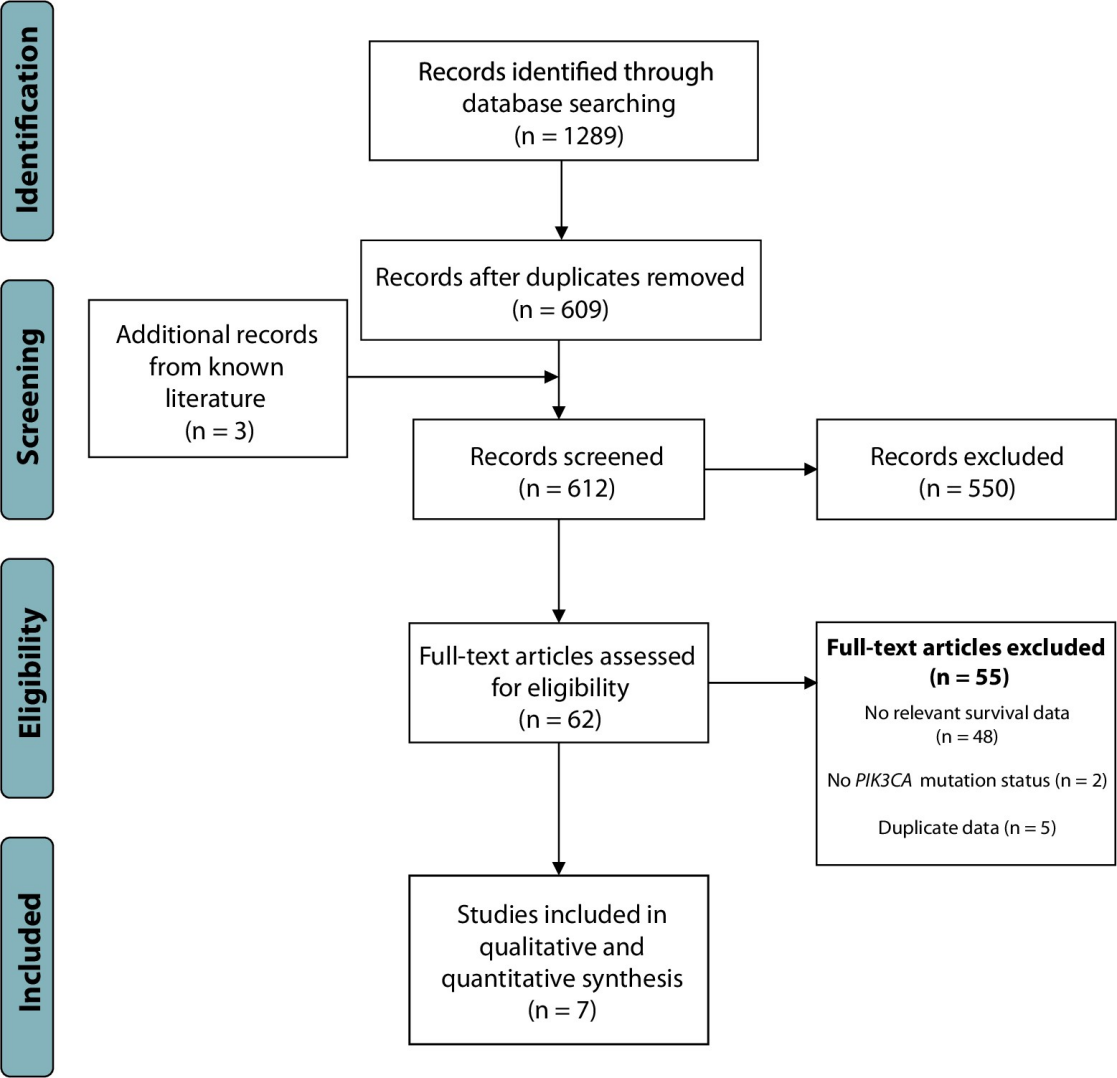
PRISMA flowchart of included and excluded literature. Studies were identified using keyword-based searches in the following databases: CENTRAL, MEDLINE, Embase and Web of Science in the primary search. In addition, publications were manually curated from the COSMIC sequencing database.

The databases CENTRAL (The Cochrane Central Register of Controlled Trials, 2008–2021), MEDLINE (Medical Literature Analysis and Retrieval System Online, 1946–2021), Embase (Excerpta Medica Database, 1974–2021) and Web of Science (1945–2021) were included in the primary search. Providers were Ovid, PubMed, Cochrane Library, and ISI web of knowledge. In addition, publications were manually curated from the COSMIC (Catalogue of Somatic Mutations in Cancer) sequencing database (2004–2021, version 94) [29]. The search employed topic-based strategies designed for each database up to 20^th^ November 2020, with no rear time limit. Language was restricted to English, with no geographical restrictions. Published conference proceedings that were included in the employed databases were also considered eligible for inclusion but were excluded if final publications were identified. The complete search strategy is presented in Fig 1 and S1 File. Updated literature searches were conducted in all mentioned databases on June 26^th^ 2022, but none of the newly released papers fulfilled the inclusion criteria.

### Data extraction

All records retrieved from the database searches were stored and managed in EndNote reference manager and Rayyan web-tool for systematic reviews [30]. Data were extracted from eligible studies that fitted the inclusion criteria by a stepwise process. First, two reviewers (H.K.B., E.A.H.) independently and blindly reviewed the titles and abstracts of all papers. Secondly, the two authors read the full text of all selected articles and excluded studies that did not meet the inclusion criteria. Thirdly, the two authors independently reviewed and extracted data from the full text articles of included studies. The following data were extracted in a standardized manner: author(s), publication year, country, study design, characteristics of the study population, follow-up time, survival data and *PIK3CA* mutation data. *PIK3CA* exon 9/20 mutations were selected for historical reasons with prior available sequencing technologies (mainly Sanger sequencing). In studies using NGS, data were extracted for *PIK3CA* exon 9/20 mutations only, to avoid bias. In studies where both OS and DSS were reported, data on DSS were extracted rather than OS. At all stages, disagreements were resolved by consensus of the authors or by a third reviewer (C.K.). When data in published papers was missing or not presented clearly, we manually curated data from online databases when possible and/or contacted authors for clarification.

### Outcome measure

The outcome measure was predefined during the design of the study as relative risk (RR) of survival rates (overall survival [OS] and/or disease-specific survival [DSS]).

### Quality assessment

Risk of bias for each included study was independently assessed by the same initial reviewers (H.K.B. and E.A.H.) using the Newcastle-Ottawa Scale, with the third reviewer (C.K.) mediating in situations of disagreement. The Newcastle-Ottawa Scale examines the risk of bias in observational studies by evaluating the selection of the study groups, the comparability of the groups, and the ascertainment of outcome of interest [31]. The summarized methodological quality of included articles was evaluated as high, medium or low using the scoring algorithm presented in S2 File.

### Statistical analysis

Statistical meta-analysis was performed with the R/RStudio version 1.3.959 using the *meta* package [32]. In a conservative approach, the random-effects model was preferred over the common-effects model since heterogeneity was anticipated, mainly because of study inclusion irrespective of stage of disease and histological subtype. Statistical heterogeneity and assessment of publication bias was assessed using I^2^, Egger’s test, Begg’s test, Thompson’s test and visual interpretation of forest plots [33–35]. An I^2^ score < 25% was considered as low heterogeneity, an I^2^ score between 25–75% as moderate heterogeneity, and an I^2^ score > 75% was considered as high heterogeneity [36]. Confidence intervals (CIs) were set at 95%. A two-sided p-value less than 0.05 was considered statistically significant.

## Results

### Literature search

A flow diagram describing the systematic literature search is given in Fig 1. The detailed search strategy for each database is given in S1 File. Initial searches yielded 1289 entries. After the removal of 680 duplicates, 609 titles and abstracts were screened. Three additional articles were then identified in already known literature, yielding a total of 62 articles that appeared relevant for inclusion. Of these, 55 articles were excluded for the following reasons: no relevant survival data (n = 48), no *PIK3CA* mutation status (n = 2) and duplicate data (n = 5).

### Included articles

This study includes 1098 patients with *PIK3CA* mutational status and survival data collected from 7 articles (Table 1) [3, 13, 14, 18, 27, 37, 38]. All studies were retrospective in design and investigated mutations of the exon 9 (helical domain) and exon 20 (kinase domain) of *PIK3CA*. One study reported data on OS while six studies reported DSS [27]. Clinical data including histologic type, grade, tumor size, tumor grade, myometrial infiltration and lymph node positivity and follow-up time are presented in Table 2. According to the criteria of the Newcastle-Ottawa scale (S2 File) [31] three of the studies were of high quality, two of medium quality, and two of low quality (Table 3).

**Table 1:** Summary of included studies.

Author (reference)	Summary of study population	Source of DNA^a^	Sequencing method^b^	Reported exons	Mutated/Total (%)	Exon 9/20 mutation ratio	Outcome measure
Bae *et al*. [37]	Patients with endometrial clear cell carcinoma, diagnosed between 2002 and 2012 were retrieved from the pathology files of four hospitals in South Korea.	FFPE	Sanger	9 and 20	3/16 (19)	2/1	DSS
Berger et al. [19]	Biospecimens collected from patients at several international institutions, diagnosed with endometrioid adenocarcinomas and serous carcinomas.	Fresh-frozen tissue	NGS	All exons	149/517 (29)	73/82	DSS
Bergstrom *et al*. [38]	Endometrioid endometrial adenocarcinomas with biopsy-proven metastatic disease, diagnosed from 2005 to 2013 at Department of Pathology, Oregon Health & Science University, USA.	FFPE	NGS	1, 4, 9, 18 and 20	6/11 (55)	2/4	DSS
Catasus *et al*. [14]	Tissue from endometrial adenocarcinomas were retrieved from the Tumor Bank of the Department of Pathology, Autonomous University, Barcelona.	Fresh-frozen tissue	Sanger	9 and 20	32/109 (29)	17/18	DSS
McIntyre *et al*. [18]	High grade endometrial carcinomas from patients diagnosed and treated between 2005 and 2011 at the Tom Baker Centre, Canada.	FFPE	Sanger	9 and 20	23/99 (23)	9/14	DSS
Mjos *et al*. [13]	Endometrial tumor samples were collected at the Department of Gynecology and Obstetrics, Haukeland University Hospital, Norway.	Fresh-frozen tissue	Sanger	9 and 20	43/278 (15)	18/26	DSS
Oda *et al*. [27]	Surgical samples from patients with endometrial carcinomas who underwent resection of their tumors at the University of Tokyo Hospital, Japan.	Fresh-frozen tissue	Sanger	9 and 20	24/66 (36)	4/20	OS

^a^ FFPE: Formalin-Fixed Paraffin-Embedded

^b^ NGS: Next generation sequencing.

**Table 2:** Characteristics of patients enrolled in included studies.

Author (reference)	Tumor size	Histologic type^a^	Grade	Myometrial infiltration F0B3 50%	Lymph node status (positive)	Follow-up time (months)
Bae *et al*. [37]	From 1.4 to 9.0cm (Mean: 3.9cm)	NEEC: 16 (CCC)	3: 16/16	na	3/12	Median 56 (Range 0–102)
Berger et al. [19]	na	EEC: 388	1: 96	na	na	Mean 38 (Range 0–225)
		NEEC: 108	2: 116			
		Mixed (EEC/SC): 21	3: 305			
Bergstrom *et al*. [38]	na	EEC: 11	1+2: 11/11	na	6/11	Median 60
Catasus *et al*. [14]	From 0.4 to 8.8cm (Mean: 4cm)	EEC: 102	1: 34/109	46/109	22/109	Mean 47 (Range 2–120)
		Mixed: 7	2: 40/109			
		(EEC/CCC 5; EEC/SC 2)	3: 35/109			
McIntyre *et al*. [18]	na	EEC: 57	3: 99/99	na	na	Mean 39 (Range 2–92)
		NEEC: 42				
Mjos *et al*. [13]	na	EEC: 228	1+2: 102/227	119/278	33/235	Median 59
		NEEC: 52	3: 174/277			
Oda *et al*. [27]	na	EEC: 58	1: 34/66	na	15/61	Mean 47
		NEEC: 8	2: 19/66			
			3: 13/66			

na; not available

^a^ EEC: Endometrioid endometrial cancer, NEEC: Non-endometrioid endometrial cancer, CCC: Clear Cell Carcinoma, SC: Serous Carcinoma

**Table 3:** Quality assessment of the included studies according to the Newcastle-Ottawa Scale.

Author, year (reference)	Selection	Comparability	Outcome	Score	Methodological quality
Bae *et al*., 2014 [37]	✩✩✩	-	✩✩✩	**6**	Low
Berger *et al*., 2018 [19]	✩✩✩✩	✩✩	✩✩	**8**	High
Bergstrom *et al*., 2016 [38]	✩✩✩✩	✩	✩✩	**7**	Medium
Catasus *et al*., 2008 [14]	✩✩✩✩	✩	✩✩	**7**	Medium
McIntyre *et al*., 2013 [18]	✩✩✩✩	✩	✩✩✩	**8**	High
Mjos *et al*., 2017 [13]	✩✩✩✩	✩✩	✩✩✩	**9**	High
Oda *et al*., 2005 [27]	✩✩✩✩	-	✩	**5**	Low

### Qualitative synthesis of survival outcomes

Oda *et al*. (2005) were the first to report survival outcomes of EC patients according to mutation status of the *PIK3CA* gene [27]. By sequencing exon 9 and -20 they found a mutation frequency of 36% and reported that *PIK3CA* mutations were more common in tumors with coexisting *PTEN* mutations. They did not observe significant differences in OS for patients with mutated *PIK3CA* gene compared to patients with wild type gene (p = 0.44) [27].

Catasus *et al*. (2008) investigated the clinicopathological impact of *PIK3CA* mutations and their relationship to other common genetic alterations in a larger series of endometrial adenocarcinomas [14]. The mutation frequency of exon 9 and -20 was 29%. They observed that *PIK3CA* mutations in exon 20 were associated with adverse prognostic features such as high grade, lymphovascular invasion and deep myometrial infiltration. However, only one of the 32 patients with *PIK3CA* mutations died from disease during a mean follow-up interval of 3.9 years [14].

McIntyre *et al*. (2013) examined the prognostic significance of *PIK3CA* mutations in 99 high-grade ECs of both endometrioid and non-endometrioid histology. They observed a statistically shorter DSS for patients with *PIK3CA* mutations, with the greatest association in grade 3 endometrioid subtypes. They also found that *PIK3CA* exon 9/20 missense mutations were more common in cases with deficient mismatch repair protein expression (p = 0.0058). The combined mutation frequency of exon 9 and -20 was 23% [18].

In 2014, Bae *et al*. sequenced *PIK3CA* in 16 pure clear cell carcinomas to evaluate clinicopathological and molecular features [37]. Three of the patients harbored mutations in *PIK3CA*, corresponding to a mutation frequency of 19%. None of the patients died of the disease during a follow up of 56 months [37].

Bergstrom *et al*. (2016) analyzed 11 pairs of primary and metastatic endometrial adenocarcinomas to compare mutational concordance between the tumors [38]. They used a semi-conductor based NGS method and found discordant *PIK3CA* mutation status between primary tumor and metastasis in 4 cases, with loss of mutation in the metastatic lesion in three cases. One of the 11 patients died of the disease during a follow-up of 60 months. This patient had a *PIK3CA* mutation conserved between the primary tumor and the metastasis. The *PIK3CA* exon 9/20 mutation frequency of in the primary tumors was 60%, being the highest reported mutation frequency among the included studies [38].

Mjos *et al*. (2017) sequenced exon 9 and exon 20 of *PIK3CA* in 280 primary ECs to assess the relationship between clinicopathologic variables and patient survival [13]. The *PIK3CA* mutation frequency of exon 9 and -20 was 15% in the primary tumors. While *PIK3CA* mutations generally had no impact on DSS (p = 0.291), exon 9 mutations were associated with poor DSS (p = 0.033 for all *PIK3CA* exon 9 mutations, p = 0.018 for charge-changing exon 9 mutations) [13]. Analysis of additional 32 metastases paired to the patient series with primary tumors demonstrated concordance between *PIK3CA* mutations and similar mutational frequencies.

Berger *et al*. (2018) performed molecular analyses of The Cancer Genome Atlas (TCGA) gynecological and breast tumor datasets, including a cohort of ECs enriched with high grade. Their study comprised the highest number of EC patients among the included studies, and extensive clinical annotation and follow-up data were available at www.cBioPortal.org, including molecular subtype [39]. The mutation frequency of exon 9 and -20 was 29%. There was no significant difference in DSS between the *PIK3CA* mutated and non-mutated groups (p = 0.193) [3].

### Quantitative analysis

#### The effect of *PIK3CA* mutations on prognosis of patients with EC

**Fig 2:**
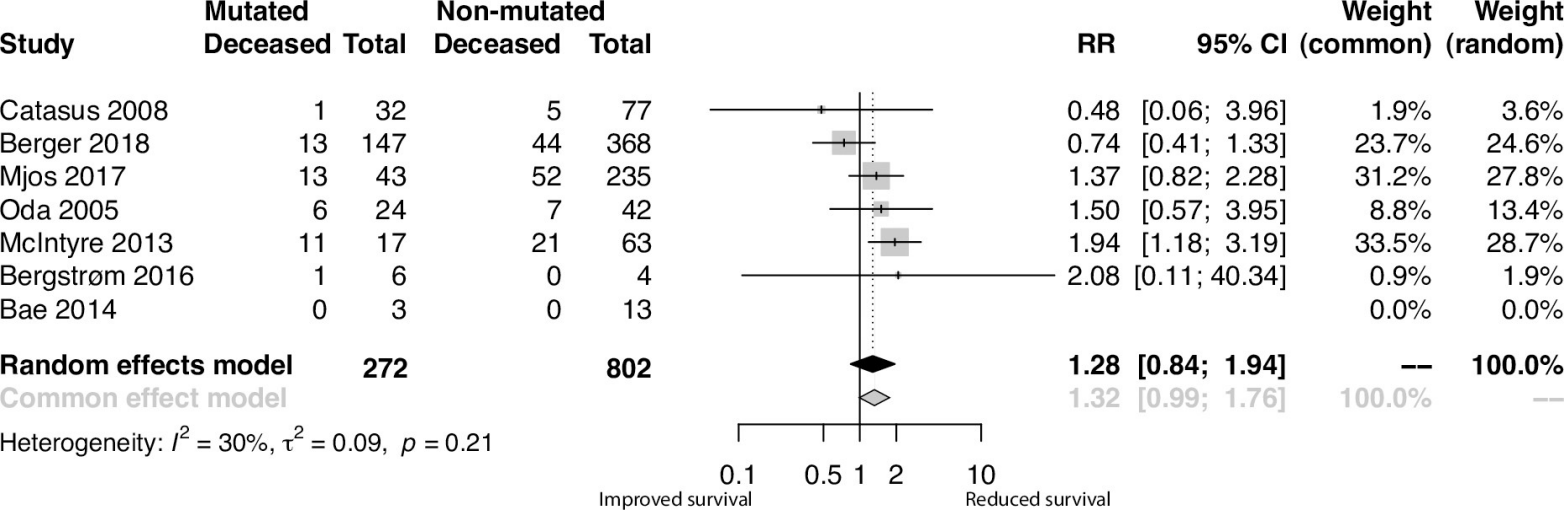
Meta-analysis results of the effect of *PIK3CA* mutations on EC patient survival. Forest plot visualizing relative risks (RR) of combined overall and disease-specific survival among studies included. Grey boxes represent relative risk of individual studies, with box size representing the inverse of the variance in individual studies and vertical black lines representing 95% confidence intervals. The diamonds represent the summarized effect of the *PIK3CA* mutation on survival for both the common effects and random effects models.

A primary meta-analysis of all included studies did not reveal any significant difference in survival outcomes between ECs with mutated and non-mutated *PIK3CA* (Fig 2; RR 1.28; 95% CI 0.84, 1.94; p = 0.25). The I^2^ score of 30% indicated that a moderate proportion of the interstudy variance was due to heterogeneity, as expected from the qualitative synthesis.

#### Subgroup meta-analyses based on histologic type

**Fig 3:**
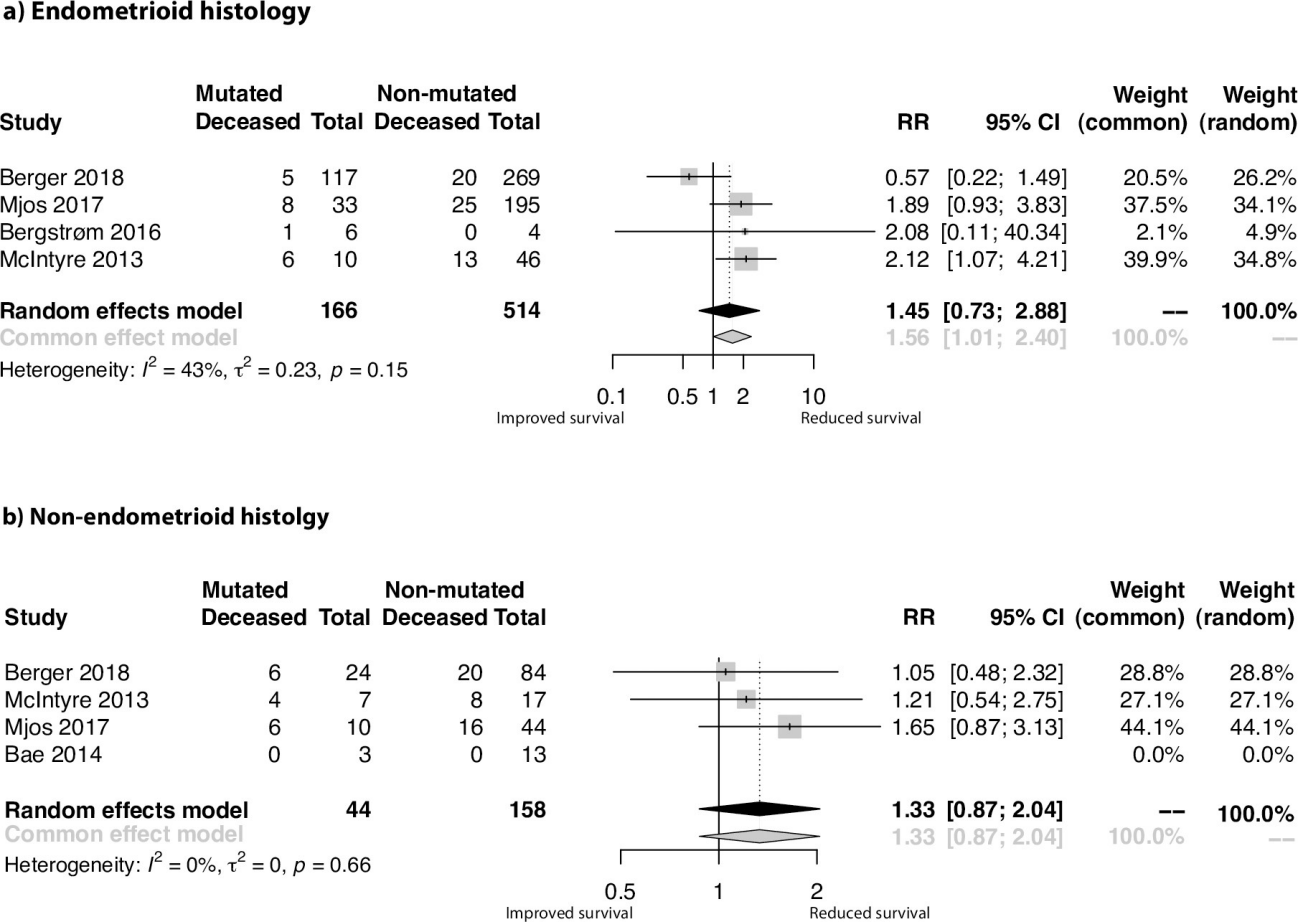
Meta-analysis results of the effect of *PIK3CA* mutations on EC patient survival related to histologic type. Forest plots of the relative risks (RR) of combined overall and disease-specific survival in **a)** endometrioid and **b)** non-endometrioid subgroups.

Four studies reported sufficient data to perform subgroup analysis on 680 ECs of endometrioid histology (EEC). The pooled RR was 1.45 (Fig 3A; 95% CI 0.73, 2.88; p = 0.29), with a tendency towards poorer survival for the EEC patient group with mutated *PIK3CA*, without reaching statistical significance. The I^2^ score of 43% suggested moderate heterogeneity in this analysis.

A total of 202 patients with non-endometrioid tumors (NEEC) were available from three studies. The pooled RR was 1.33 (Fig 3B; 95% CI 0.87, 2.04; p = 0.18) and the null I^2^ score indicated no interstudy variance.

#### Subgroup meta-analyses based on tumor grade

**Fig 4:**
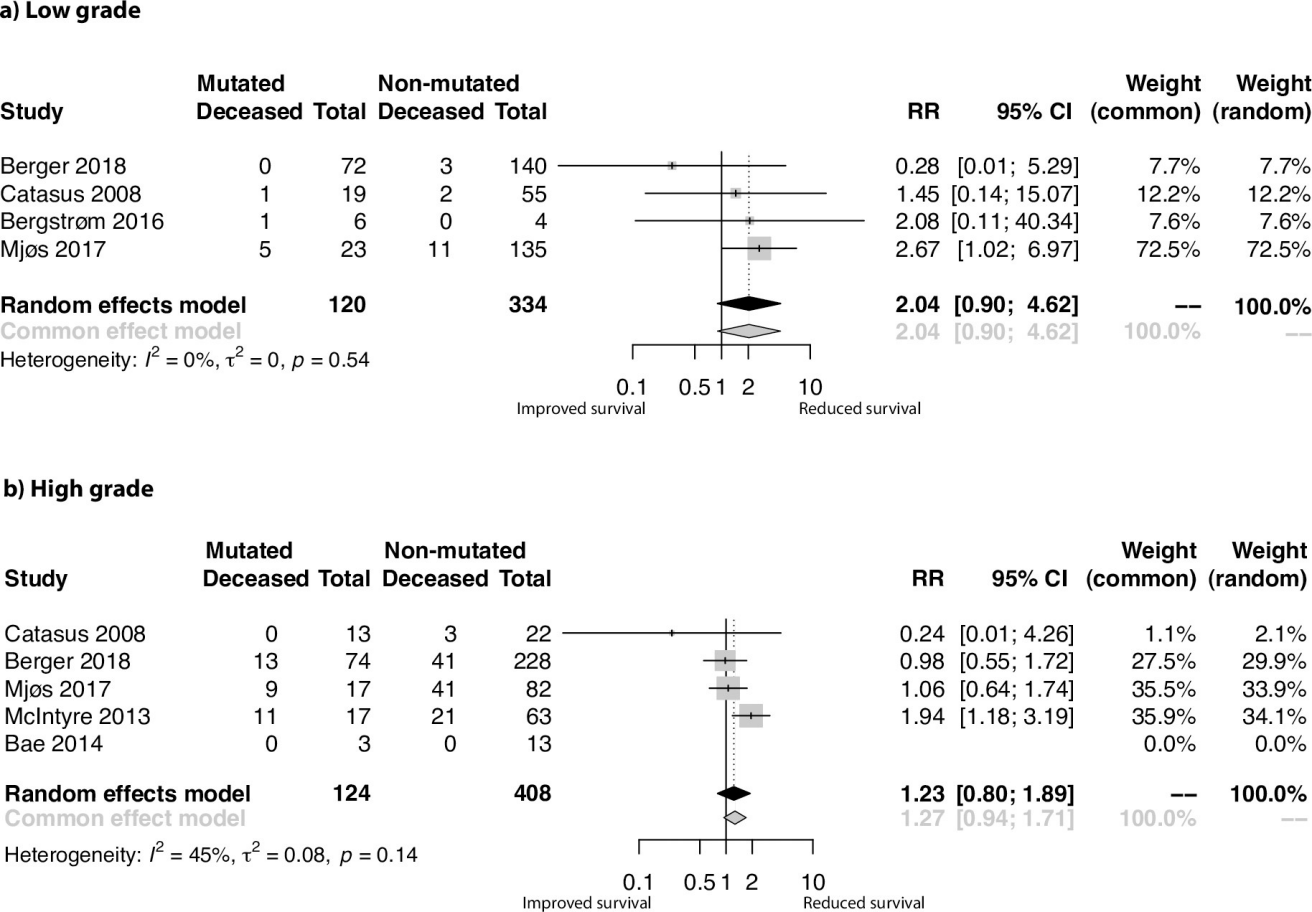
Meta-analysis results of the effect of *PIK3CA* mutations on EC patient survival in relation to grade. Forest plots visualizing relative risks (RR) of combined overall and disease-specific survival in the **a)** low-grade and **b)** high grade subgroups.

For analysis within low-grade disease, a total of 454 patients from four studies were analyzed. The I^2^ score of 0% indicated that no interstudy variance was due to heterogeneity. The pooled RR was 2.04 (Fig 4A; 95% CI 0.90, 4.62; p = 0.09), suggesting a doubled risk of poor outcome in the *PIK3CA* mutated group, reaching borderline significance.

Four studies reported data about 532 patients with high grade disease. The pooled RR was 1.23 (Fig 4B); 95% CI 0.80, 1.89; p = 0.34) and the I^2^ score of 45% revealed that a moderate proportion of the interstudy variance was due to heterogeneity.

### Estimation of publication bias

Risk of publication bias was evaluated with Egger’s test, supplemented by Begg’s test and Thompson’s test [33–35], as multiple tests are recommended when few studies are available in meta-analysis [40]. The result showed no evidence of publication bias in any of the tests (Egger’s test; p = 0.70, Begg’s test; p = 0.85, Thompson’s test; p = 0.73.), supported by visual interpretation of the funnel plot with a symmetrical distribution of studies (S1 Fig) [41]. However, as few studies are available, the power of these methods is low [42].

## Discussion

Seven eligible studies with *PIK3CA* exon 9/20 mutation rates ranging from 19–55% were included in this meta-analysis to assess the impact of *PIK3CA* mutations on survival outcomes in EC. We specifically focused on mutations of exon 9 and exon 20 in *PIK3CA*. These regions account for majority of the total *PIK3CA* mutational burden detected by NGS, including hotspot mutations in the helical- and kinase domains, and have commonly been target regions of *PIK3CA* in Sanger sequencing approaches.

In our primary analysis including all seven studies, *PIK3CA* exon 9/20 mutation status had no significant impact on survival outcomes of patients with EC. Hence, the reported *PIK3CA* mutations in EC, despite a high mutation frequency, do not contribute considerable to the clinicopathologic phenotypes. We note moderate heterogeneity in our qualitative analysis that could be due to differences in the observed *PIK3CA* mutational status. This was evident in the range of mutation frequencies reported and may be explained by variation in sequencing technology applied, the source of tissue available for DNA isolation, or inter- and intra-cohort differences in FIGO stages and molecular classes. As presented in Table 1, the included studies used DNA extracted from both FFPE and fresh-frozen tissue for DNA sequencing. Fresh frozen tissue remains the preferred source for DNA, as artifacts may arise from the FFPE-fixation procedure. Concerning FIGO staging, all studies included ECs of all stages, but data were insufficient to stratify *PIK3CA* mutation status according to FIGO stage. Altogether, the variations in the eligible studies appear to explain our observed moderate heterogeneity in the primary analysis, motivating additional subgroup analyses.

Subgroup analyses were performed for histologic type and grade with no significant association of *PIK3CA* exon 9/20 mutations with survival. The null I^2^ scores in low-grade and NEEC subgroup-analysis show low interstudy heterogeneity, strengthening the confidence of these results.

All our analysis, both primary and subgroup analysis, indicated a tendency (non-significant) of reduced survival for patients with mutated *PIK3CA*. The strongest effect was observed in the subgroup analysis of low-grade ECs with border line significance (p = 0.09). This could suggest that the effect of exon9/20 *PIK3CA* mutations on survival depends on the mutational context, possibly influenced by factors such as co-mutations in other genes. The effect of *PIK3CA* mutations may be more potent in low-grade tumors because these tumors usually have a higher mutation burden with increased potential for synergistic effects, e.g. multi-mechanistic PI3K activation [43], whereas *PIK3CA* mutations may be less potent in high-grade ECs where survival already is poorer and the effect may be overpowered by features such as copy-number alterations [3].

The location of the *PIK3CA* mutation may have clinical relevance. In the study by Catasus *et al*., *PIK3CA* exon 9 mutations were more common in low-grade tumors while exon 20 mutations more common in high-grade tumors [14]. Other studies have suggested that there are differences in patient survival depending on the exon-specific location of the *PIK3CA* mutations, as both individual helical (exon 9) or kinase domain (exon 20) mutations have been associated with adverse prognostic effects in EC [13, 14]. *In vitro* experiments have demonstrated that mutations in exons 9 or exon 20 can transform cancer cells through different and independent signaling pathways [44]. Hotspot mutations in the helical domain of exon 9 affects a surface patch of the p110α protein and changes its ability to interact with other regulatory proteins, whereas hotspot mutations in exon 20 affect the kinase domain in proximity to the hinge region of the catalytic loop leading to constitutive activation [14, 45]. Among the studies included in this report, *PIK3CA* exon 20 mutations were more frequent than exon 9 mutations in four of the studies [13, 18, 27, 38], evenly distributed in two of the studies [3, 14] and with lower occurrence in one study [37]. Interestingly, the two studies reporting evenly distributed mutation frequencies between exon 9 and exon 20 were the two studies suggesting favorable outcome for the mutated genotype in the primary analysis [3, 14]. Hence, stratifying patients according to the location of *PIK3CA* mutations in larger cohorts is suggested to potentially reveal underlying effects on survival. Unfortunately, individual patient data regarding location of mutations, FIGO stages and the recent new molecular classification (as suggested by TCGA) was insufficient to perform such subgroup analyses in this study.

Forthcoming studies should include full length mutation data and complete clinical annotations to facilitate proper patient stratification and to elucidate the role of *PIK3CA* in survival. Such data should also include race/ethnicity for the evaluation of differences in *PIK3CA* mutation rates that has been observed in EC and that might be relevant for optimal patient treatment in different countries [46].

There are some limitations that dampens the conclusions from our meta-analysis. First, the number of studies that assessed the relationship between *PIK3CA* mutations and survival of patients with EC was limited, with only seven studies available. Second, the size of the study populations varied considerably among the different studies. Small studies with few patients are often a source of publication bias and are more likely to introduce confounding results than larger studies as this reduces the statistical power of the pooled results [47]. However, the present study also has several important strengths. Risk of publication bias was evaluated using three independent tests where none showed evidence of publication bias. In addition, none of our meta-analyses was interpreted as high level of heterogeneity; the primary analysis was evaluated to intermediate heterogeneity, while the subgroup analysis was evaluated as low-intermediate (NEEC and Grade^Low^; low heterogeneity, EEC and Grade^High^; intermediate heterogeneity). In addition, assessment of the qualities of studies included in the meta-analysis using the Newcastle-Ottawa scale revealed that our analyses in large are based on papers of sufficient quality. To our knowledge, this study is the most powerful and comprehensive synthesis of evidence on this issue to date with a total sample size of 1098 patients.

## Conclusions

In summary, this meta-analysis based on current literature indicates a tendency that *PIK3CA* mutations in exon 9 and/or exon 20 have a negative effect on survival of patients with EC. This tendency was most prominent in patients with low-grade tumors, suggesting that these patients may favor *PIK3CA* testing and potential targeted therapy the most.

### Statement of ethics

This systematic review leverages on literature already published as individual studies. Survival data were extracted from publications as anonymous data, and hence no institutional review board approval was required.

## Supporting information

S1 Checklist PRISMA 2009 checklist. (PDF)

S1 File Search strategy. (DOCX)

S2 File Modified Newcastle-Ottawa quality assessment scale and scoring algorithm. (DOCX)

S1 Fig Funnel plot. Funnel plot of the estimation of publication bias on the effect of *PIK3CA* exon 9 and exon 20 mutations on survival outcomes of endometrial cancer patients.
(DOCX)
