# Supplementary material for: PIK3CA mutations and their impact on survival outcomes of patients with endometrial cancer: A systematic review and meta-analysis
Source: PLoS One. 2023 Mar 21;18(3):e0283203. doi: 10.1371/journal.pone.0283203 (PMC10030019; doi:10.1371/journal.pone.0283203)
Supplement: S1 File — (DOCX) [file pone.0283203.s002.docx]

**S1 file: Search strategy**

**PubMed**

"((endometrial cancer[All Fields] AND (((""PIK3CA""[All Fields] OR ""PI3K""[All Fields]) OR ""p110a""[All Fields]) OR ""phosphatidylinositol-4,5-bisphosphate 3-kinase catalytic subunit alpha""[All Fields])) AND (survival)) AND (mutation)",Most Recent,,"((((((""endometrial neoplasms""[MeSH Terms] OR (""endometrial""[All Fields] AND ""neoplasms""[All Fields])) OR ""endometrial neoplasms""[All Fields]) OR (""endometrial""[All Fields] AND ""cancer""[All Fields])) OR ""endometrial cancer""[All Fields]) AND (((""PIK3CA""[All Fields] OR ""PI3K""[All Fields]) OR ""p110a""[All Fields]) OR ""phosphatidylinositol-4,5-bisphosphate 3-kinase catalytic subunit alpha""[All Fields])) AND ((((((((((""mortality""[MeSH Subheading] OR ""mortality""[All Fields]) OR ""survival""[All Fields]) OR ""survival""[MeSH Terms]) OR ""survivability""[All Fields]) OR ""survivable""[All Fields]) OR ""survivals""[All Fields]) OR ""survive""[All Fields]) OR ""survived""[All Fields]) OR ""survives""[All Fields]) OR ""surviving""[All Fields])) AND ((((((((((""mutate""[All Fields] OR ""mutated""[All Fields]) OR ""mutates""[All Fields]) OR ""mutating""[All Fields]) OR ""mutation""[MeSH Terms]) OR ""mutation""[All Fields]) OR ""mutations""[All Fields]) OR ""mutation s""[All Fields]) OR ""mutational""[All Fields]) OR ""mutator""[All Fields]) OR ""mutators""[All Fields])"

**Results:** 103

**MEDLINE**

| **#** | **Searches** | **Results** |
| --- | --- | --- |
| 1 | ("endometri* cancer*" or "endometri* carcinoma*" or "endometri* neoplasm*" or "cancer of endometrium" or "cancer of the endometrium").ti,ab,kw. | 26651 |
| 2 | endometrium cancer/ or endometrium tumor/ or endometrium carcinoma/ | 22746 |
| 3 | (PIK3CA or p110a or "p110-a" or p110alpha or "p110-alpha" or "phosphatidylinositol-4,5-bisphosphate 3-kinase catalytic subunit alpha").ti,ab,kw. | 5746 |
| 4 | mutat*.ti,ab,kw. | 689276 |
| 5 | exp mutation/ | 788973 |
| 6 | 1 or 2 | 33395 |
| 7 | 4 or 5 | 1112034 |
| 8 | 3 and 6 and 7 | 216 |

**EMBASE**

| **#** | **Searches** | **Results** |
| --- | --- | --- |
| 1 | endometrium cancer/ or endometrium tumor/ or endometrium carcinoma/ | 56049 |
| 2 | ("endometri* cancer*" or "endometri* carcinoma*" or "endometri* neoplasm*" or "cancer of endometrium" or "cancer of the endometrium").ti,ab,kw. | 40332 |
| 3 | exp mutation/ | 6679 |
| 4 | mutat*.ti,ab,kw. | 925745 |
| 5 | PIK3CA or p110a or "p110-a" or p110alpha or "p110-alpha" or "phosphatidylinositol-4,5-bisphosphate 3-kinase catalytic subunit alpha").ti,ab,kw. | 12687 |
| 6 | 1 or 2 | 62391 |
| 7 | 3 or 4 | 1436662 |
| 8 | 5 and 6 and 7 | 529 |

**CENTRAL**

| **#** | **Searches** | **Results** |
| --- | --- | --- |
| 1 | (Endometrial cancer) AND PIK3CA | 17 |

**COSMIC**

| **#** | **Searches** | **Results** |
| --- | --- | --- |
| 1 | (Endometrial cancer) AND PIK3CA | 5 |

**Web of Science**

| **#** | **Searches** | **Results** |
| --- | --- | --- |
| 1 | Endometri* AND PIK3CA | 419 |
