# Supplementary material for: PIK3CA mutations and their impact on survival outcomes of patients with endometrial cancer: A systematic review and meta-analysis
Source: PLoS One. 2023 Mar 21;18(3):e0283203. doi: 10.1371/journal.pone.0283203 (PMC10030019; doi:10.1371/journal.pone.0283203)
Supplement: S2 File — (DOCX) [file pone.0283203.s003.docx]

**S2 file: Modified Newcastle-Ottawa quality assessment scale and scoring algorithm**

Note: A study can be given a maximum of one star for each numbered item within the Selection and Outcome categories. A maximum of two stars can be given for Comparability.

**Selection**1) Representativeness of the exposed cohort
a)  Truly representative (one star)
b)  Somewhat representative (one star)
c)  Selected group
d)  No description of the derivation of the cohort

2)  Selection of the non-exposed cohort
a)  Drawn from the same community as the exposed cohort (one star)
b)  Drawn from a different source
c)  No description of the derivation of the non-exposed cohort

3)  Ascertainment of exposure
a)  Secure record (e.g., surgical record) (one star)
b)  Structured interview (one star)
c)  Written self report
d)  No description
e)  Other

4)  Demonstration that outcome of interest was not present at start of study
a) Yes (one star)
b) No

**Comparability**1) Comparability of cohorts on the basis of the design or analysis controlled for confounders
a)  The study controls for age (one star)
b)  Study controls for grade (one star)
c)  Cohorts are not comparable on the basis of the design or analysis controlled for confounders

**Outcome**1)  Assessment of outcome
a)  Independent blind assessment (one star)
b)  Record linkage (one star)
c)  Self report
d)  No description
e)  Other

2)  Was follow-up long enough for outcomes to occur
a) Follow-up 3 years or more (one star)
b) Follow-up less than 3 years

3) Adequacy of follow-up of cohorts
a)  Complete follow up - all subject accounted for (one star)
b)  Subjects lost to follow up unlikely to introduce bias - number lost less than or equal to 20% or description of those lost suggested no different from those followed. (one star)
c)  Follow up rate less than 80% and no description of those lost
d)  No statement

**Thresholds for converting the Newcastle-Ottawa scores to high, medium and low methodological quality:**

**High quality:** 3 or 4 stars in selection domain AND 1 or 2 stars in comparability domain AND 2 or 3 stars in outcome/exposure domain

**Medium quality:** 2 stars in selection domain AND 1 or 2 stars in comparability domain AND 2 or 3 stars in outcome/exposure domain

**Low quality:** 0 or 1 star in selection domain OR 0 stars in comparability domain OR 0 or 1 stars in outcome/exposure domain
