# Supplementary material for: PIK3CA mutations and their impact on survival outcomes of patients with endometrial cancer: A systematic review and meta-analysis
Source: PLoS One. 2023 Mar 21;18(3):e0283203. doi: 10.1371/journal.pone.0283203 (PMC10030019; doi:10.1371/journal.pone.0283203)
Supplement: S1 Fig — Funnel plot of the estimation of publication bias on the effect of PIK3CA exon 9 and exon 20 mutations on survival outcomes of endometrial cancer patients. (DOCX) [file pone.0283203.s004.docx]

**S1 fig: Funnel plot**

**S1 fig:** Funnel plot of the estimation of publication bias on the effect of *PIK3CA* exon 9 and exon 20 mutations on survival outcomes of endometrial cancer patients.
